# A New Art

**Published:** 1888-06-23

**Authors:** 


### A New Art.

HITHERTO something has been done in the direction of plating non-conducting surfaces, as may be seen by the heads of many of the modern canes and umbrellas, by coating them with plumbago, but this is of necessity a somewhat imperfect and unsatisfactory process, except for the rudest work. A Frenchman, however, substitutes a bath, the composition of which is a secret, for the plumbago, and into which objects to be electro-plated are plunged for a few seconds. When withdrawn they are seen to be coated with a delicate bloom, like that of a plum, which enables them to readily receive any coating of metal which may be desired. A beautiful example of this art is a large decorated panel of roses, buds, violets, smilax, and other flowers and foliage, and the whole is apparently most beautifully carved in bronze. In it the delicate curves and veinings of the smallest petals and leaves are wrought with marvellous fidelity, and it holds the spectator in doubt as to where nature ends and art begins. In point of fact the whole thing is produced in a few hours, and each bronze flower and leaf contains the original upon which the metal has been deposited by electric action, and it is asserted that flowers so encased retain not only their natural colours, but that their perfumes are also entombed for ever. This same process is also applied to making silver flies, beetles, and other insects, and has progressed into the realm of the lizard and small snake when needed for purposes of decoration. Just where this process may end it is difficult to say, and as the animal so hermetically sealed will never decay, it needs but a little stretch of the imagination to hear ourselves saying with Shakespeare, "My father in his habit as he lived," on examining the curios of some wealthy individual, who for reasons of his own may have thus embalmed his nearest relative.

